# Dependable Performance of Thin Film Composite Nanofiltration Membrane Tailored by Capsaicin-Derived Self-Polymer

**DOI:** 10.3390/polym14091671

**Published:** 2022-04-20

**Authors:** Yuanyuan Tang, Lu Cao, Li Xu, Zhaoyu Wang, Qian Shi, Yingying Zhang, Liangmin Yu

**Affiliations:** 1 Institute of Oceanographic Instrumentation, Qilu University of Technology (Shandong Academy of Sciences), Shandong Provincial Key Laboratory of Ocean Environmental Monitoring Technology, National Engineering and Technological Research Center of Marine Monitoring Equipment, Qingdao 266100, China; tyytougao@163.com (Y.T.); caolu@qlu.edu.cn (L.C.); t.31@163.com (Z.W.); srdwjm@sina.com (Q.S.); 2 Key Laboratory of Marine Chemistry Theory and Technology, Ministry of Education, Ocean University of China, Qingdao 266100, China; xuli2022@126.com; 3 Open Studio for Marine Corrosion and Protection, Pilot National Laboratory for Marine Science and Technology, Qingdao 266100, China

**Keywords:** nanofiltration membrane, capsaicin derivatives, self-polymer, interfacial polymerization, desalination

## Abstract

To address trade-off and membrane-fouling challenges during the development of nanofiltration membranes, a thin-film composite membrane was prepared on the basis of interfacial polymerization regulated by adjusting the capsaicin-derived self-polymer poly N-(2-hydroxy-5-(methylthio) benzyl) acrylamide (PHMTBA) on the polysulfone substrate in this study. Through the self-polymerization of the monomer HMTBA with varied contents, microwave-assisted technology was employed to develop a variety of PHMTBAs. It was discovered that PHMTBA is involved in the interfacial polymerization process. Piperazine and PHMTBA competed for the reaction with trimesoyl chloride, resulting in a flatter and looser membrane surface. The PHMTBA-modified membrane presented a typical double-layer structure: a thicker support layer and a thinner active layer. The addition of PHMTBA to membranes improved their hydrophilicity and negative charge density. As a result, the PHMTBA-modified membrane showed dependable separation performance (water flux of 159.5 L m^−2^ h^−1^ and rejection of 99.02% for Na_2_SO_4_) as well as enhanced anti-fouling properties (flux recovery ratio of more than 100% with bovine serum albumin-fouling and antibacterial efficiency of 93.7% against *Escherichia coli*). The performance of the prepared membranes was superior to that of most other modified TFC NF membranes previously reported in the literature. This work presents the application potential of capsaicin derivatives in water treatment and desalination processes.

## 1. Introduction

With population growth and the exacerbation of water pollution, the freshwater resource crisis has emerged as one of the most pressing worldwide challenges. Among a variety of solutions, membrane separation technology, which has the advantages of high separation efficiency and low energy consumption, shows excellent potential for water treatment [1,2]. Among them, nanofiltration (NF) membranes with pore sizes ranging from 0.5 nm to 2 nm have a separation capacity between that of ultrafiltration (UF) and reverse osmosis membranes. The NF membrane separation process is achieved by combining the size exclusion and Donnan exclusion separation mechanisms and is conducive to application in seawater desalination [3]. Thin-film composite (TFC) membranes with high flux and separation selectivity are made up of a thin polyamide (PA) selective layer and a thicker support layer, which is advantageous in the NF process. Membrane fouling, on the other hand, remains one of the most severe obstacles to the practical applications of TFC NF membranes. As a result, it is critical to mitigate membrane fouling to preserve water permeability, lower membrane operation costs, and enhance the competitiveness of TFC NF membranes [4,5].

Several methods for improving membrane fouling have been proposed in the literature, such as grafting hydrophilic polymers (polyethylene glycol, polydopamine, and zwitterions) onto the membrane surface [6,7] and introducing inorganic nanomaterials (titanium dioxide, carbon nanotubes, and metal-organic framework) into the membrane [8,9,10]. These methods can improve membrane hydrophilicity and limit the adsorption between the contaminants and the membrane surface to decrease membrane-fouling propensity [11]. Despite these merits, the increased membrane thickness from the grafting layer may raise extra mass transfer resistance during the NF process. Moreover, nanoparticles with complex preparation processes increase the cost of the membranes and the difficulty of large-scale preparation. In addition, the grafted materials or nanomaterials may be released during long-term operation, affecting membrane performance. Thus, advanced hydrophilic materials with better compatibility and compactness need to be developed to prepare TFC NF membranes with improved desalination performance and anti-fouling properties.

Capsaicin, the main active ingredient in chili peppers, is a type of vanillin amide alkaloid with phenolic hydroxyl [12]. Capsaicin and its derivatives have been applied in painting ocean hulls because of their good anti-fouling and antibacterial properties [13]. Capsaicin and its derivatives have also recently been used in membrane separation to improve membranes’ anti-fouling properties [14,15]. Wang et al. [16] blended the capsaicin-derived monomer N-(2-hydroxy-3-butyl-4-methyl) acrylamide into a polyethersulfone (PES) UF membrane, which improved its water flux and antibacterial properties. Considering the aforementioned findings, capsaicin and its derivatives are potential materials for membrane water treatment. In addition, Xu et al. [17] used an in situ polymerization method during the blending process to prepare polysulfone (PSf) membranes containing the capsaicin-derived self-polymer poly N-(2-hydroxy-3-methyl acrylamide-4,6-dimethyl benzyl) acrylamide. The resulting membrane showed more than twice the pure water permeability (PWP) of the virgin membrane and was more resistant to humic acid (HA) fouling. However, because of the lack of strong chemical bonds and self-crosslinking with the membrane bulk polymer, simply blended capsaicin derivatives may gradually separate from the membrane matrix during the filtration process, affecting the anti-fouling performance and mechanical stability of the membrane [18]. Therefore, our group [19] proposed a new method in which the capsaicin mimic-monomer propyl 2-(acrylamidomethyl)-3,4,5-trihydroxybenzoate (PAMTB) was incorporated in the PA layer to participate in the interfacial polymerization (IP) of a TFC NF membrane. The membrane modified with PAMTB exhibited a 43% increase in water flux and approximately 100% permeation flux recovery after HA fouling. However, NF membranes modified by capsaicin derivatives face two issues. First, the capsaicin-derived monomer is easily oxidized because of the existence of an unsaturated bond [17], resulting in membrane instability. Second, the complete self-polymerization of the capsaicin derivative is difficult to achieve using traditional polymerization methods (e.g., hydrothermal polymerization) because phenolic hydroxyl acts as a capping agent in the molecules [20,21]. A new efficient polymerization method needs to be developed to make the most of the capsaicin derivative during the membrane preparation process.

Compared with the conventional heating method, microwave-assisted (MWA) technology, with the advantages of straightforward operation, short reaction time, and low energy consumption, has been widely employed in chemical synthesis as a multifunctional module platform because of its straightforward operation, short reaction time, and low energy consumption [22,23,24,25]. MWA technology is currently being used for the polymerization of capsaicin derivatives because of its higher polymerization efficiency. Yu et al. [26] successfully constructed an anti-fouling composite PSf/PES membrane via the in situ polymerization of the capsaicin derivative (5-methyl acrylamide-2,3,4 hydroxy benzyl) acrylamide with MWA technology.

In this work, the capsaicin-derived self-polymer poly N-(2-hydroxy-5-(methylthio) benzyl) acrylamide (PHMTBA) was synthesized by the self-polymerization of single-monomer HMTBA without any additional monomers via MWA technology, a process that differed from the co-polymerization of multiple monomers in the previous literature [13,14]. The modified TFC NF membrane was created with the self-polymer PHMTBA by one-step IP into the PA layer. The PHMTBA and piperazine (PIP) in the aqueous phase solution competed for the reaction with trimesoyl chloride (TMC), precisely tailoring the membrane surface morphology and properties. Moreover, the effects of PHMTBA on permeability and selectivity were systematically investigated. Furthermore, the anti-fouling properties of PHMTBA-modified NF membranes, including organic fouling and antibacterial activity, were also evaluated. For the first time, this research explored the desalination behavior of the capsaicin-derived self-polymer on TFC NF membranes, and it provides a promising way to construct a novel TFC NF membrane with superior performance. 

## 2. Materials and Methods

### 2.1. Materials

**Figure 1:**
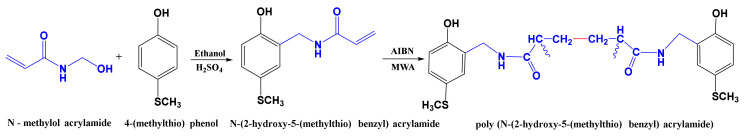
The synthesis route of self-polymer PHMTB.

Commercial PSf UF membranes were purchased from Lenntech Water Treatment Inc. (Beijing, China). HMTBA was prepared according to Figure 1, and the characterization data were analyzed as described in the Supporting Information. 2,2′-Azodiisobutyronitrile (AIBN) was used as the initiator and ethanol was used as the solvent to promote the self-polymerization of HMTBA (Note S1 from the Supplementary Materials). PIP (>99.5%, J&K Scientific, Beijing, China) and TMC (>99.0%, J&K Scientific, Beijing, China) were used as the monomers to form the PA layer via the IP reaction. The model organic pollutants were used as the feed solution to test the anti-organic fouling property. Inorganic salts, including Na_2_SO_4_, MgCl_2_, MgSO_4_, and NaCl (2 g/L) were utilized to measure the selectivity of the prepared membranes. All the chemical reagents except for those with special instructions were commercially available from Sinopharm Chemical Reagent Co. Ltd. (Shanghai, China) without further purification. Deionized (DI) water produced by the Millipore Milli-Q Advantage A10 system (Molsheim, France) was used throughout this work.

### 2.2. Self-Polymer PHMTBA Synthesis

The literature [27,28] has reported the synthesis route of the self-polymer PHMTBA, as shown in Figure 1. In detail, 0.6 mol *N*-methylol acrylamide (MA) and 0.5 mol 4-(methylthio) phenol (MTP) were dissolved together in ethanol. Then, concentrated H_2_SO_4_ (~10 mL) as a catalyst was added dropwise under continuous stirring conditions to keep the reaction temperature at about 35 °C. The reaction was maintained for 72 h. Afterward, the obtained product was purified via filtering, centrifugation, multiple washing, and recrystallization until the product became nearly neutral. The white powder of the monomer HMTBA was obtained. Then, a certain amount of the monomers HMTBA and AIBN (at a ratio of 5:1), which were dissolved in ethanol, was added to a specialized tests tube. The reaction temperature was raised to 80 °C and held for 5 min by microwave irradiation at 144 W in a single-mode microwave reactor (CEM Discover SP, CEM, Matthews, NC, USA). The self-polymer PHMTBA was synthesized in a uniform and homogeneous solution.

### 2.3. Membrane Fabrication

**Figure 2:**
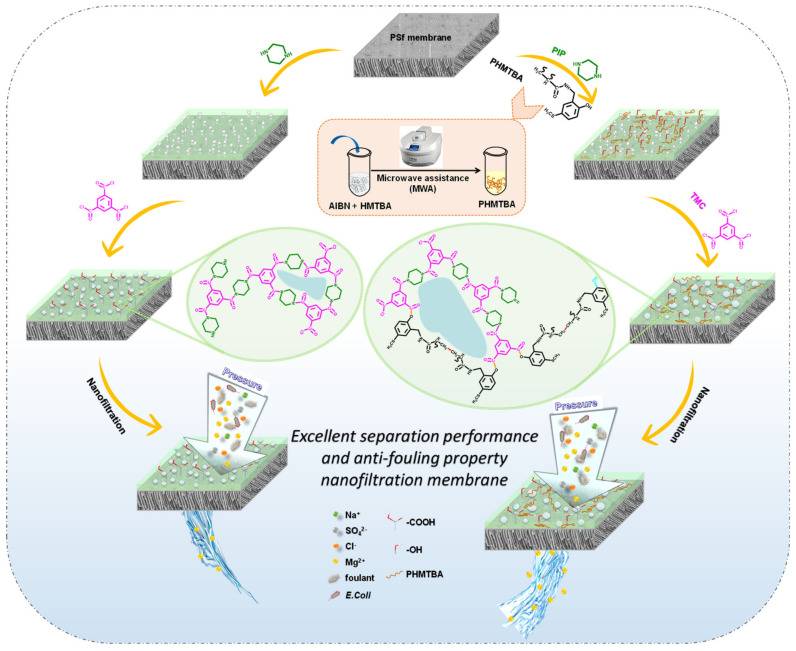
The schematic diagram of a fabrication process of unmodified and PHMTBA-modified TFC membranes.

PHMTBA-modified TFC membranes were designed using a typical one-step IP method [27], as displayed in Figure 2. The PSf support layer made contact with a 2% (*w*/*w*) aqueous phase solution consisting of a PHMTBA ethanol solution and a PIP aqueous solution. After 2 min, the excess aqueous phase solution was removed from the substrate surface with a rubber roller. Subsequently, 0.1% (*w*/*v*) TMC *n*-hexane solution made contact with the substrate for 30 s. After the excess organic solution was taken out, the PHMTBA-modified membrane was cured at a temperature of 80 °C, which was held for 5 min by a drier. Finally, the prepared membranes were stored in DI water at least overnight before testing.

The prepared membranes were labeled as PA-PHMTBA_X_, where the x represents the initial HMTBA concentration of 0–0.1% (*w*/*w*).

### 2.4. Membrane Characterization

The chemical properties of the PHMTBA-modified membranes were measured by attenuated total reflectance Fourier transform infrared spectroscopy (FTIR, Tensor 27, Bruker, Karlsruhe, Germany), X-ray photoelectron spectroscopy (XPS, ESCALAB 250XI, Thermo Fisher Scientific, Waltham, MA, USA), and thermogravimetry (TG, STA449 F3, NETZSCH, Selb, Germany). The surface morphology of the TFC membranes was captured by scanning electron microscopy (SEM, S-4800, Hitachi, Tokyo, Japan). The water contact angle (WCA) was estimated with a contact angle meter (DSA 100, Kruss, Heidelberg, Germany) to characterize the hydrophilicity. The surface charge property was measured with a zeta potential analyzer (SurPASS III, Anton Paar, Ashland, VA, USA) in streaming potential mode using a test solution of 0.001 mol L^−1^ KCl, in which pH values were adjusted from 3 to 10 by 0.05 mol L^−1^ NaOH and 0.05 mol L^−1^ HCl solutions. Zeta potential values were recorded with the pH variation.

### 2.5. Membrane Performance 

Membrane performance was measured by a cross-flow filtration system (FlowMem00024-PN20, Fumei, Xiamen, China) at 0.5 MPa. The velocity was 7.5 L min^−1^. The operation temperature was held constant with the help of circulating water at room temperature. The effective filtration area of the membrane was 24 cm^2^.

#### 2.5.1. Separation Performance

For the purpose of stability considerations, the membranes were pre-compacted for 50 min at 0.6 MPa before each measurement. Permeance (*PWP*, L/m^2^hbar) was calculated according to the Equation (1):

(1) $$PWP=\frac{Q}{A_{m}\Delta t\Delta P}$$

where Q (L) is the volume of the permeate solution, $A_{m}$ (m^2^) is the effective membrane area, (Δt) is the collection time, and ΔP (MPa) is the transmembrane pressure difference.

Rejection (*R*, %) was measured using 2.0 g/L Na_2_SO_4_, MgCl_2_, MgSO_4_, and NaCl as the feed solutions. The conductivity of the feed solutions and permeate solutions was measured with a digital conductivity meter (DDS-307A, INESA, Shanghai, China). *R* was calculated using the Equation (2):

(2) $$R=\left(1-\frac{C_{p}}{C_{f}}\right)\times 100\%$$

where $C_{p}$ and $C_{f}$ represent the salt concentrations in the permeate and feed solutions, respectively. All tests were conducted in triplicate for the effectiveness of the data, and the average of the results was taken.

In addition, the stability of the PHMTBA-modified membranes was tested over a prolonged period of time with Na_2_SO_4_ solution as the feed solution, and the normalized flux and rejection were determined with the time variation.

#### 2.5.2. Anti-Fouling Property

The anti-fouling property was investigated on the basis of organic fouling and biological fouling resistance. Organic fouling experiments were conducted using HA, BSA, and SA (0.5 g/L) as the representative pollutants under the same cross-flow filtration system and operation parameters. Normalized flux was employed with a consistent initial water permanence for a visualized comparison. The test protocol was implemented according to the reported literature, including four main steps [19,28]. First, the membranes were filtered with DI water to record the first pure water flux ($F_{0}$); second, the membranes were continuously filtered, substituting the model organic foulant solution for DI water to record the first permeate flux ($F_{1}$) for 60 min; third, the membranes were re-filtered with DI water after rinsing with DI water for 30 min to record the second pure water flux ($F_{2}$); and fourth, the second stage was repeated to record the second permeate flux ($F_{3}$). The fouling indexes were used to estimate the anti-organic fouling properties, namely, the flux recovery ratio (FRR, %), the total flux decline ratio ($R_{t}$, %), the reversible fouling ratio ($R_{r}$, %), and the irreversible fouling ratio ($R_{ir}$, %), which are defined as the Equations (3)–(6):

(3) $$FRR=\frac{F_{2}}{F_{0}}\times 100\%$$

(4) $$R_{t}=\left(1-\frac{F_{1}}{F_{0}}\right)\times 100\%$$

(5) $$R_{r}=\left(\frac{F_{2}-F_{1}}{F_{0}}\right)\times 100\%$$

(6) $$R_{ir}=\left(\frac{F_{0}-F_{2}}{F_{0}}\right)\times 100\%=R_{t}-R_{r}$$

The biofouling resistance of the PHMTBA-modified membrane was quantitatively analyzed on the basis of the literature, with *E. coli* bacterial cells as the model microorganism [29,30]. Briefly, *E. coli* was cultured with Luria-Bertani (LB) liquid medium, followed by shaking for 12 h at 37 °C. The membrane samples (unmodified and modified membranes) were sterilized with 75% (*v*/*v*) alcohol ahead of time. Then these samples were placed into tubes and immersed in a diluted fresh *E. coli* suspension. Then, they were kept at 37 °C for 24 h in an incubator. Sterilized saline solution was used to collect the cells. The collected solution was poured onto an LB agar plate with a triangular glass coating rod after it was diluted to a predetermined concentration. The treated LB agar plate was incubated for another 24 h at 37 °C to estimate colony formation. To minimize experimental errors, each test was conducted in triplicate. *E. coli* colonies with uniform growth were counted to calculate the antibacterial efficiency of each sample (r, %) according to the plate count method [31], as shown in Equation (7):

(7) $$r=\frac{w_{t}-Q_{t}}{w_{t}}\times 100\%$$

where $w_{t}$ and $Q_{t}$ are the numbers of bacterial colonies on the surfaces of unmodified and PHMTBA-modified TFC membranes, respectively.

## 3. Results

### 3.1. Characterizations

#### 3.1.1. Membrane Chemical Property

**Figure 3:**
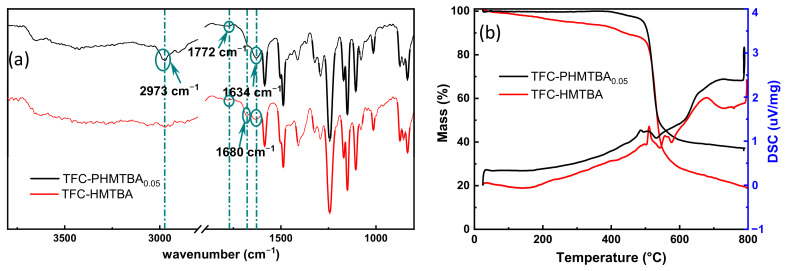
(**a**) FTIR spectra and (**b**) TG curve of PA−PHMTBA and PA−HMTBA membranes.

To probe the successful modification of TFC membranes by PHMTBA, the chemical structure of the membrane was characterized with FTIR spectra, as depicted in Figure 3a. The characteristic peak at 1634 cm^−1^ for C=O stretching vibration represented the amide bands from the typical IP reaction between PIP and TMC. In addition, the weaker peak at 1772 cm^−1^ for C=O stretching vibration from the ester bands was caused by the reaction between the hydroxyl in the capsaicin derivative and the acyl chloride in TMC, demonstrating the successful participation of the capsaicin derivative in the IP process. Furthermore, the stretching vibration peak at 2973 cm^−1^ for -CH_2_ was discovered in the PA-PHMTBA_0.05_ membrane, which was associated with the polymerization of C=C. Compared with the PA-HMTBA membrane, the intensity of the absorption peak for C=C at 1680 cm^−1^ of the PA-PHMTBA_0.05_ membrane became weaker, consistent with the FTIR spectrum of PHMTBA powder (Figure S1). This finding demonstrated that the polymer PHMTBA was involved in the IP reaction to modify the PA layer successfully.

TG analysis was used to detect the thermal properties of the PA layer in Figure 3b. In general, a similar mass loss trend for PA-PHMTBA_0.05_ and PA-HMTBA membranes was observed. In detail, the mass for both membranes decreased sharply between 500 °C and 600 °C and leveled off when the temperature continued to rise, which indicated that the primary PA material for the PA layer stayed the same with the introduction of a capsaicin derivative in the form of a monomer or a polymer [32]. Upon further observation, the mass decline of the PA-PHMTBA_0.05_ membrane began at about 400 °C, while that of the PA-HMTBA membrane occurred from the onset of heating. Moreover, the PA-PHMTBA_0.05_ membrane displayed a higher mass percentage after stabilization, which is ascribed to the more stable PA layer with the modification of the capsaicin-derived polymer PHMTBA with more alkyl chains and fewer unsaturated bonds (Figure 3a).

**Figure 4:**
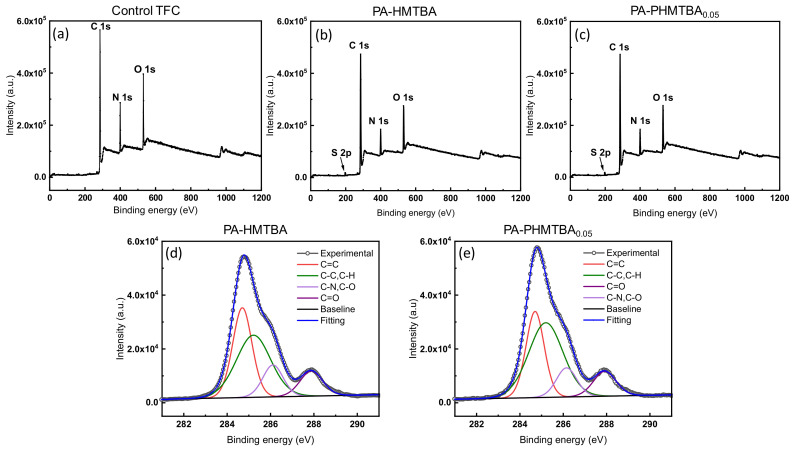
XPS assessment of as-fabricated membranes. XPS spectra of (**a**) control TFC membranes, (**b**) PA-HMTBA membranes, and (**c**) PA-PHMTBA membranes; C1s survey spectra of (**d**) PA-HMTBA and (**e**) PA-PHMTBA membranes.

The element composition of the as-prepared membrane was characterized by the XPS spectrum. As shown in Figure 4a–c, the XPS wide-scan spectra of membranes displayed three main peaks at 284.8 eV of C1s, 399.7 eV of N1s, and 531.0 eV of O1s. Compared with the control TFC membrane, a new peak, S2p, emerged in the PA-HMTBA and PA-PHMTBA membranes as a result of the successful participation of capsaicin derivatives in the modified membrane. Table 1 lists the atomic compositions and O/N ratios of the different membranes. The modified membranes showed higher C contents and O/N ratios compared with the control TFC membrane, which may be attributed to the participation of capsaicin derivatives, resulting in increasing alkyl chains. To further explore the possible surface chemical compositions of the modified membranes, the C1s spectra of the PA-PHMTBA_0.05_ and PA-HMTBA membranes were curve-fitted into four peaks: C=C, at about 284.7 eV [33]; C-C and C-H, at about 285.2 eV; C=O, at about 287.9 eV; and C-N and C-O, at about 286.1 eV (Figure 4d,e). The existence of the C-N characteristic peak indicated that the amide bond formed via an IP process. Compared with the PA-HMTBA membrane, the composition of the reduced C=C peak and the rising C-H peak of the PA-PHMTBA_0.05_ membrane (Table 1), owing to the formation of several C-H bonds [26], indicated the introduction of the polymer PHMTBA into the PA layer. Another detailed analysis of the PA layer formula in Note S2 and Figure S2 confirmed the successful participation of PHMTBA in the cross-linked network of the PA layer, which was consistent with the FTIR spectrum results (Figure 3a). However, the C-S peak could not be found in the C1s survey spectra, which may be because of the lower content of sulfur.

#### 3.1.2. Membrane Morphological Characterizations

**Figure 5:**
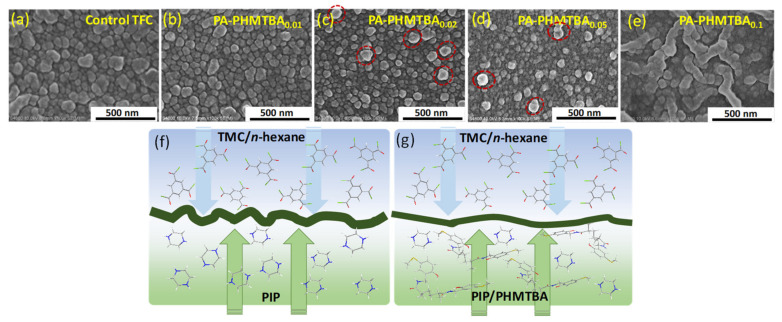
(**a**–**e**) SEM surface images and (**f**,**g**) schematic diagrams of the interfacial diffusion process of unmodified and PHMTBA-modified TFC membranes. (The red coils in (**c**,**d**) represented the particle-like structure).

The surface morphologies of developed membranes with different amounts of PHMTBA are shown in Figure 5a–e. Overall, the modified TFC membrane presented the typical double structure, which was composed of a PSf support layer and a PA layer (Figure S3). All membrane surfaces displayed raised and nodular structures, which are typical structural features of a PA layer formed by PIP and TMC via an IP reaction [34,35]. With the increase in the PHMTBA concentration from 0% to 0.05%, the membrane showed a more particle-like and uneven PA layer with a decreased size of the nodular structure. This is because the aqueous phase solution diffusion from the support layer to the organic phase was hampered by the addition of long-chain PHMTBA (Figure 5f,g). Moreover, the less dense primary PA layer resulted from the decreased solubility of PHMTBA as the polymer concentration increased (Note S3 and Figure S4), which also influenced the surface structure. All PHMTBA-modified membrane surfaces also presented varying degrees of nano-tubular Turing structure (Figure S5), in particular the PA-PHMTBA_0.1_ membrane (Figure 5e) [36]. It was inferred that the PHMTBA molecule, containing a large number of hydroxyl groups, might limit the diffusion of the aqueous phase monomer to the interface because of the hydrogen bonding force. Moreover, the excessive long-chain molecules in the aqueous phase solution were prone to a wrinkled structure in the PA layer, or the role of PIP/PHMTBA was the same as that of the activator. This was conducive to improving the permeance, which will be discussed later.

#### 3.1.3. Membrane Surface Properties

**Figure 6:**
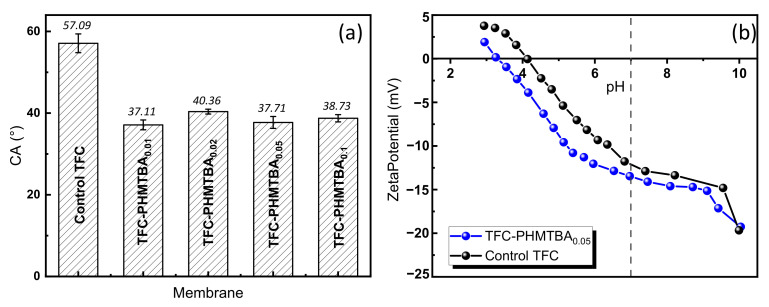
(**a**) WCA of unmodified and PHMTBA−modified membranes and (**b**) zeta potential at different pH values of control TFC and PA−PHMTBA_0.05_ membranes.

Figure 6a shows the WCA results of the prepared TFC membranes for characterizing the hydrophilicity. The WCA exhibited a decrease from the highest 57.1° of the control TFC membrane [37] to 37.1° of the PA-PHMTBA_0.01_ membrane, indicating an improvement in membrane hydrophilicity. However, the WCA of the modified membranes with increased PHMTBA contents remained almost unchanged, suggesting that the hydrophilicity of the PHMTBA-modified membrane was independent of the PHMTBA concentration. The increased alkyl chain resulting from a higher PHMTBA concentration in the aqueous phase might negatively influence hydrophilicity. Moreover, the decreased solubility of PHMTBA with high concentrations in the PIP/PHMTBA solution (Figure S4) limited the fixation of the aqueous phase monomers on the substrate surface, affecting the further improvement of the hydrophilicity. Despite this, the addition of PHMTBA contributed to the hydrophilicity of the modified membrane.

As expressed by the Donnan theory, the selectivity of TFC NF membranes can be affected by the surface electrical properties expressed by the zeta potential [38]. As shown in Figure 6b, the PA-PHMTBA_0.05_ and control TFC membranes were both negatively charged when the pH was higher than 4, which was due to the hydrolysis of excess -C=O-Cl groups in the PA layer. Furthermore, the PA-PHMTBA_0.05_ membrane showed a stronger negative charge density than the control TFC membrane at all pH values because of the introduction of abundant hydroxyl in PHMTBA. Especially under neutral conditions, the PHMTBA-modified TFC membrane was favorable for anion rejection and fouling resistance.

### 3.2. Optimization of PHMTBA-Modified Membranes

#### Separation Performance

**Figure 7:**
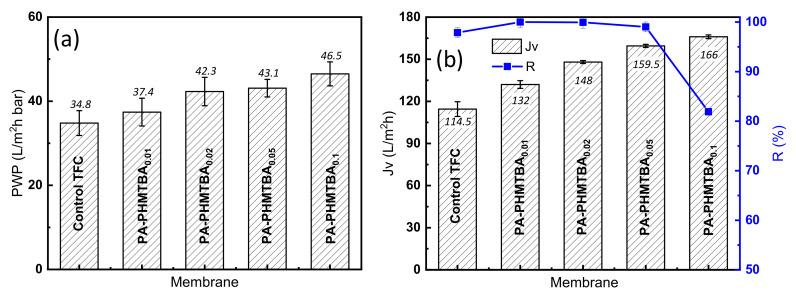
(**a**) Permeance and (**b**) selectivity of prepared TFC membranes.

As discussed above, the prepared TFC membranes featured a hydrophilic, even, and electronegative surface induced by PHMTBA as modifiers involved in the IP process, which was beneficial for superior permeance and rejection. From Figure 7a, the permeance of the TFC membranes gradually increased with the increase in PHMTBA concentration. The membrane surface structure (Figure 5 and Figure S5) contributed to the result. The permeate flux of the membranes showed a similar trend as the permeance and reached a maximum of 166.0 L/m^2^h with the highest PHMTBA concentration (Figure 7b). This is probably because of the improved hydrophilicity of modified TFC membranes (Figure 6a). The rejection of the Na_2_SO_4_ solution for the PHMTBA-modified membrane was slightly increased with a PHMTBA concentration below 0.05% (*w*/*w*). The negative charge density of the membrane surface played an important role (Figure 6b). However, the rejection for the PA-PHMTBA_0.1_ membrane was dramatically decreased, which was related to the wrinkled surface structure. Therefore, the PA-PHMTBA_0.05_ membrane was selected as the representative PHMTBA-modified membrane with optimum performance (a relatively high water flux of 159.5 L/m^2^h and a satisfactory rejection of 99.02%) for the next test.

### 3.3. Performances of the Optimized PHMTBA-Modified Membranes

#### 3.3.1. Selectivity for Different Salts and Membrane Stability

**Figure 8:**
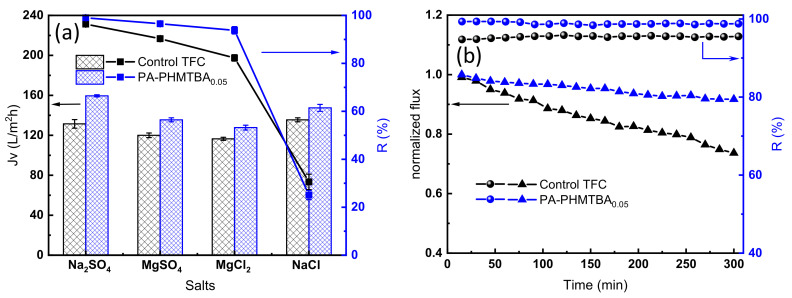
(**a**) Selectivity for different inorganic salt solutions and (**b**) stability of the control TFC membrane and the PA-PHMTBA_0.05_ membrane.

The ion selectivity was predicted by measuring the separation performance of the membrane with different monovalent and divalent inorganic salts as feed solutions (Figure 8a). The PA-PHMTBA_0.05_ membrane showed greater permeate flux than the control TFC membrane in all salt solutions because of its improved hydrophilicity. The PA-PHMTBA_0.05_ membrane exhibited outstanding rejection performance compared with the control membrane except when NaCl was used as the feed solution, which can probably be ascribed to the more negatively charged surface yet looser pore structure, simulated in Figure 2. The rejection of the PA-PHMTBA_0.05_ membrane for different inorganic salts followed the order Na_2_SO_4_ (99.0%) > MgSO_4_ (96.5%) > MgCl_2_ (93.8%) > NaCl (25.1%). On the one hand, the negatively charged membrane surface (Figure 6b) signified a larger repulsion capacity for divalent anions (SO_4_^2−^ > Mg^2+^ > Cl^−^), and in order to maintain the charge balance in the bulk solution, the rejection of MgSO_4_ was lower than that of Na_2_SO_4_. On the other hand, because of the larger hydration radius (Table S1) and the slower diffusion rate, the rejection of Mg^2+^ was higher than that of Na^+^. Thus, it could be confirmed that the separation selectivity of the PHMTBA-modified NF membrane was determined by size-sieving and Donnan exclusion [39]. In summary, the TFC membrane modified by the capsaicin-derived polymer PHMTBA indicates the potential selectivity of divalent and monovalent salts.

The membrane stability was compared to expand the application prospects, and the results are shown in Figure 8b. The flux decline of the PA-PHMTBA_0.05_ membrane was less than 10%, and the rejection was maintained above 99%, exhibiting stable performance under long-term operation. However, a large flux decline was found for the control TFC membrane. This indicates that the essential stability of the polymerized PHMTBA and its involvement in PA layer formation (Figure 2) could indeed be beneficial for the operation stability of the modified membrane.

#### 3.3.2. Anti-Fouling Properties

**Figure 9:**
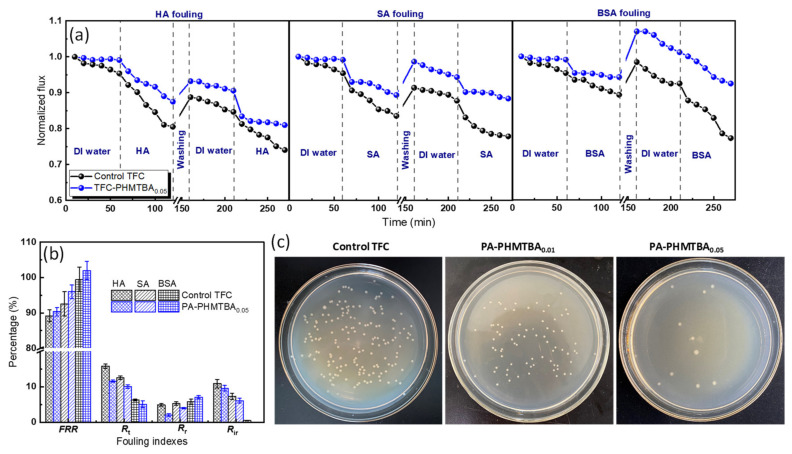
Anti-fouling properties of the as-prepared TFC membrane. (**a**) Normalized fluxes with various pollutants filtration, (**b**) fouling indexes, and (**c**) images of Petri dishes of the unmodified and PHMTBA-modified membranes.

The excellent anti-fouling properties, including organic fouling and biofouling, are beneficial for broadening the application potential, prolonging the lifespan, and reducing the operation costs of membranes [40]. On the basis of its performance, the organic fouling tendency of the PA-PHMTBA_0.05_ membrane was analyzed using different model pollutants in Figure 9a,b. For comparison, the control TFC membrane was also investigated as a reference. A similar flux variation trend of membranes with or without PHMTBA was observed. In the first phase, the pure water flux was maintained relatively constant with DI water filtration. In the second phase, the permeate flux declined obviously with the pollutants as the feed solutions. In the third phase, the pure water flux recovered to a large extent after simple hydraulic washing. In the fourth phase, the permeate flux greatly decreased because of repeated contamination. Compared with that of the unmodified and modified membranes, the organic fouling of the PA-PHMTBA_0.05_ membrane was alleviated (Figure 9a) thanks to its more negatively charged surface, which exhibited higher flux recovery and lower fouling indexes irrespective of pollutant type (Figure 9b). For example, with regard to HA fouling, the FRR and $R_{t}$ of the PA-PHMTBA_0.05_ and control membranes were 90.4% and 89.2%, respectively, and 11.6% and 15.8%, respectively. The detailed fouling indexes also indicated that the hydrophilic surface could endow the membrane with strong organic fouling resistance. However, the PHMTBA-modified membrane exhibited diverse organic fouling behaviors related to the intrinsic qualities of various pollutants. For instance, the $R_{r}$ values of the PA-PHMTBA_0.05_ membrane for HA, SA, and BSA were 2.0%, 3.1%, and 7.1%, respectively. The organic fouling extent of the prepared TFC membrane for HA was the most severe with the highest $R_{ir}$ index of 10.9%. This may be ascribed to the strong binding affinity between HA and the membrane surface [41,42]. Surprisingly, the PA-PHMTBA_0.05_ membrane fouled by BSA exhibited an FRR of more than 100% (Figure 9b), indicating that BSA was easily resisted and released with simple washing for the TFC membrane modified with PHMTBA.

Several studies have investigated the antibacterial properties of capsaicin and its derivatives so that they can be used in membrane preparation [13,17,43,44]. However, most studies have focused on the effects of the monomers or copolymers on the UF process. Here, the effect of the capsaicin-derived self-polymer PHMTBA on the antibacterial property of TFC NF membranes was investigated, and the images of Petri dishes are shown in Figure 9c. The number of colonies on the membrane surface was significantly reduced after the modification of PHMTBA. Because of the strong inhibitory effect on the growth and reproduction of bacteria [45], the PA-PHMTBA_0.05_ membrane exhibited an antibacterial efficiency of 93.7% against *E. coli* (details in Table 2). The improved surface hydrophilicity also played a part. The efficient antibacterial properties could alleviate the biofouling problem of the PHMTBA-modified NF membrane system.

### 3.4. Performance Comparison

Table 3 shows a performance comparison between the as-prepared membrane in this work and other reported NF membranes. According to the experimental results, the PA-PHMTBA membranes in this work exhibited much better separation performance, with one of the best permeances and the most competitive rejection. Meanwhile, the PA-PHMTBA membranes exhibited the highest FRR after organic fouling. The PA-PHMTBA membranes exhibited better performance compared with other NF membranes. This finding demonstrates that the capsaicin-derived self-polymer could endow the TFC membrane with superior separation performance and anti-fouling properties through the manipulation of the structure of the PA layer. This work offers a prospective way to design NF membranes with enhanced performance for the water treatment process.

## 4. Conclusions

This work developed a novel TFC NF membrane wherein the synthetic capsaicin-derived self-polymer PHMTBA constructed via MWA technology was introduced into the membrane selective layer. Various characterizations demonstrated that as a comonomer, PHMTBA in the aqueous phase solution successfully participated in the IP reaction. In comparison to the control membrane, the modified membrane presented a flatter surface and a stronger membrane surface negative charge density. Meanwhile, the hydrophilicity of the modified membrane was improved by the addition of PHMTBA with functional groups. The superior separation performance and anti-fouling properties of the PHMTBA-modified membrane were enhanced as a result of these features. The modified TFC membrane with a 0.05% (*w*/*w*) PHMTBA concentration showed an increased water flux of 159.5 L/m^2^h and a rejection of more than 99%. Meanwhile, the PHMTBA-modified membrane exhibited excellent anti-organic fouling capacity for HA, SA, and BSA and 93.6% antibacterial efficiency. The performance of the PA-PHMTBA membranes was superior to that of the other NF membranes reported in the literature. This study presents the application potential of capsaicin derivatives in the preparation of high-performance TFC membranes for the desalination process.

## Figures and Tables

**Table 1:** XPS surface elemental compositions and high-resolution C1s spectra of the prepared membranes.

Membrane	Atomic Contents (%)				Atomic Ratio	Species Contents (%)			
	C	O	N	S	O/N	C=C	C-C, C-H	C=O	C-N, C-O
Control TFC	72.64	13.12	14.24	/	0.92	/	/	/	/
PA-HMTBA	80.45	10.69	8.52	0.34	1.25	34.06	41.99	11.22	12.74
PA-PHMTBA_0.05_	80.96	10.36	8.36	0.32	1.24	29.60	48.47	10.69	11.23

**Table 2:** Antibacterial property of the as-prepared TFC membrane.

Membrane	CFU after Incubating for 24 h	Antibacterial Efficiency (%)
Control TFC	158 ± 6	/
PA-PHMTBA_0.01_	22 ± 4	53.4
PA-PHMTBA_0.05_	10 ± 1	93.7

**Table 3:** Performance comparison of prepared membrane with other NF membranes reported in the literature.

Membrane	Permeance (L/m^2^hbar)	R (%)	FRR (%)	Testing Conditions	Ref.
PIP/ZA-TMC	29.0	91.0	95.2	6 bar, 1.0 g/L MgSO_4_, 1.0 g/L BSA (2 bar)	[3]
TFN-SGO	2.37	96.5	98.0	5 bar, 2.5 g/L Na_2_SO_4_, 0.25 g/L BSA in 0.025 g/L Na_2_SO_4_	[10]
TFC-PAMTB	~27.5	98.0	~92.0	5 bar, 2.0 g/L Na_2_SO_4_, 0.5 g/L BSA	[19]
AAIL-TFC	12.2	94.5	/	6 bar, 1.0 g/L MgSO_4_	[27]
M-40COOH	48.1	84.0	40.1	6 bar, 1.0 g/L Na_2_SO_4_, 0.5 g/L BSA	[28]
PA/GO TFN	1.47	~97.0	/	15 bar, 2.0 g/L MgSO_4_	[38]
TFC-cGO	~11.66	99.2	96.0	7 bar, 2.0 g/L MgSO_4_, 1.0 g/L BSA	[40]
NFM-PAO	25.2	~99.0	76.0	4 bar, 1.0 g/L Na_2_SO_4_, 1.0 g/L BSA + 1.0 g/L Na_2_SO_4_	[46]
PDA@SiO_2_-PMIA	31.3	97.0	/	6 bar, 1.0 g/L Na_2_SO_4_	[47]
TFN-AA/GO	11.34	>95.0	/	8 bar, 1.0 g/L Na_2_SO_4_	[48]
TFC-HPE	50.62	98.0	95.7	2 bar, 1.0 g/L Na_2_SO_4_, 0.5 g/L HA	[49]
TFC	34.8	97.8	99.5	5 bar, 2.0 g/L Na_2_SO_4_, 0.5 g/L BSA	This work
PA-PHMTBA	43.1	99.0	>100.0	5 bar, 2.0 g/L Na_2_SO_4_, 0.5 g/L BSA	This work

## Data Availability

Not applicable.

## Supplementary Materials

The following supporting information can be downloaded at https://www.mdpi.com/article/10.3390/polym14091671/s1, Figure S1: (a) ^1^H NMR spectra with DMSO-d6 as the solvent, (b) TG curves of monomer HMTBA, and (c) FTIR spectra of HMTBA and PHMTBA; Figure S2: Theoretical molecular formulas of interfacial polymerization among PIP, PAMTB, and TMC; Figure S3: The cross-section structure of the PA-PHMTBA_0.05_ membrane; Figure S4: Photos of the PIP/PHMTBA aqueous phase solution; Figure S5: Local SEM surface images of unmodified and PHMTBA-modified TFC membranes; Table S1: The hydrated radius of different ions. Reference [50] is cited in supplementary materials.
