# Supplementary material for: Dependable Performance of Thin Film Composite Nanofiltration Membrane Tailored by Capsaicin-Derived Self-Polymer
Source: Polymers (Basel). 2022 Apr 20;14(9):1671. doi: 10.3390/polym14091671 (PMC9103837; doi:10.3390/polym14091671)
Supplement: Supplementary file 1 [file polymers-14-01671-s001.zip › polymers-1672454-supplementary.pdf]

polymers

MDPI

Supplementary Materials:

Dependable Performance of Thin Film Composite Nanofiltration Membrane Tailored by Capsaicin-Derived Self-polymer

Yuanyuan Tang [1], Lu Cao [1], Li Xu [2,3], Zhaoyu Wang [1], Qian Shi [1], Yingying Zhang [1,*] and Liangmin Yu [2,3,*]

Institute of Oceanographic Instrumentation, Qilu University of Technology (Shandong Academy of Sciences), Shandong Provincial Key Laboratory of Ocean Environmental Monitoring Technology, National Engineering and Technological Research Center of Marine Monitoring Equipment, Qingdao, 266100, China; tyytougao@163.com (Y.T.); caolu@qlu.edu.cn (L.C.); t.31@163.com (Z.W.); srdwjm@sina.com (Q.S.)

2 Key Laboratory of Marine Chemistry Theory and Technology, Ministry of Education, Ocean University of China, Qingdao, 266100, China; 1033505249@qq.com (L.X.); yuyan@ouc.edu.cn (L.Y.)

3 Open Studio for Marine Corrosion and Protection, Pilot National Laboratory for Marine Science and Technology, Qingdao, 266100, China

* Correspondence: yuyan@ouc.edu.cn (L. Yu); triciayyz@163.com (Y. Z.)

Note S1: Characterizations

The monomer HMTBA structure was characterized by ^{1}H NMR spectra (^{1}H NMR, Avance III HD, Broker, Germany), FTIR spectra (FTIR, Tensor 27, Broker, Germany), and TG curves (TGA, STA449 F3, NETZSCH, Germany), shown in Figure S1. ^{1}H NMR spectra (Figure S2a) was resolved as follows. δ: 2.37 (s, 3H, CH_3), 4.25 (d, 2H, CH_2), 5.63 (m, 1H, CH=), 6.14 (m, 1H, $=CH_2$), 6.29 (m, 1H, $=CH_2$), 6.80 (m, 1H, PhH), 7.08 (m, 2H, PhH), 8.50 (s, 1H, NH), and 9.67 (s, 1H, OH) and was coincident with the structural formula (Figure 1). The TG curves (Figure S1b) characterized the stability of HMTBA, where the obvious weight loss began at above 200 °C. The clear distinctions between FTIR spectra of monomer HMTBA and polymer PHMTBA are displayed in Figure S2c. The peaks at 2920 cm^{-1} and 2851 cm^{-1} representing sp2 -C-H and sp3 -C-H stretching vibrations, respectively, only appeared for PHMTBA, verifying the successful attachment of long-chain alkyl. In addition, the peak of C=C stretching at 1680 cm^{-1} was weaker in PHMTBA than that in HMTBA, which signified the successful polymerization of monomer HMTBA.

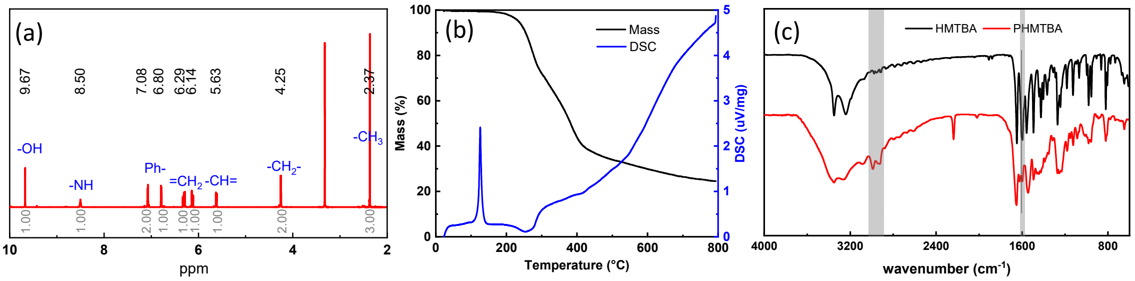

Figure S1. (**a**) ^{1}H NMR spectra with DMSO-d6 as the solvent, (**b**) TG curves of monomer HMTBA, and (**c**) FTIR spectra of HMTBA and PHMTBA.

Note S2:

Assuming that the IP reaction occurred between PIP and TMC, the molecular formulas of the PA layer in the control TFC membrane were $C_7H_8ON_2$ and $C_{13}H_{13}O_5N_2$ with fully cross-linked and fully linear patterns, respectively (Figure S2a and c). The responding ratios of O/N were 0.57 and 2.86, respectively. For the PA-PHMTBA membrane, it was hypothesized that the IP process reacted only between PHMTBA and TMC, the molecular formulas of the PA layer were $C_{25}H_{24}O_5N_2S_2$ and $C_{31}H_{29}O_9N_2S_2$ with fully cross-linked and fully linear patterns, respectively (Figure S2b and d). The responding ratios of O/N were

2.86 and 5.14, respectively. Thus, it was inferred that the ratio of O/N should be increased when PHMTBA competed with PIP to react with TMC during IP process. The results in Table 1 revealed the increased atomic ratio of O/N for PA-PHMTBA membrane contrast with the control TFC membrane, consistent with the inference mentioned above. The participation of PHMTBA in the cross-linked network of the PA layer was demonstrated.

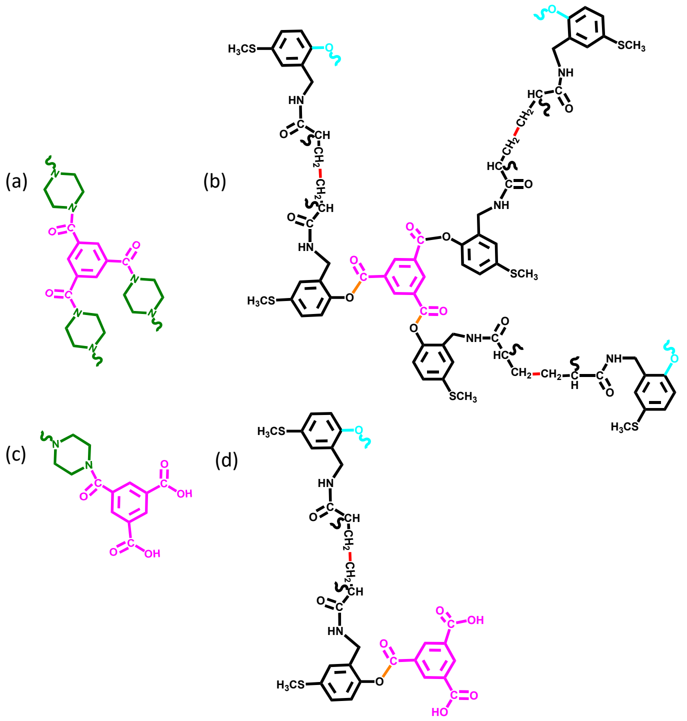

Figure S2. Theoretical molecular formulas of interfacial polymerization among PIP, PAMTBA, and TMC. (**a**) Fully cross-linked reaction between PIP and TMC, (**b**) fully cross-linked reaction between PAMTBA and TMC, (**c**) fully linear reaction between PIP and TMC and (**d**) fully linear reaction between PAMTBA and TMC.

Note S3:

The SEM and TEM cross-section morphologies of the PA-PHMTBA0.05 membrane presented the typical double structure, which was composed of the PSf support layer and PA layer. The thickness of the PA layer was about 33 nm, which was measured by the Image-Pro Plus 6.0 software.

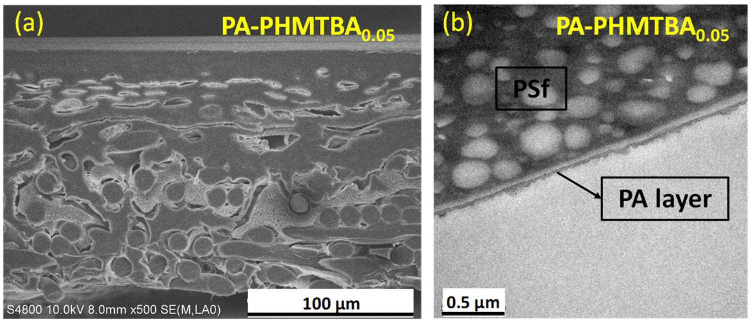

Figure S3. The (**a**) SEM and (**b**) TEM cross-section morphology of PA-PHMTBA0.05 membrane.

Note S4:

The aqueous phase solutions including PIP and PHMTBA with 0 to 0.1% and the polymerized PHMTBA ethanol solution were photographed and are displayed in Figure S3. With the increase in PHMTBA concentration, the solubility of PHMTBA decreased,

and the PHMTBA was precipitated. The color of the aqueous phase solution changed from a limpid solution to a faint yellow solution with more concentrated PHMTBA.

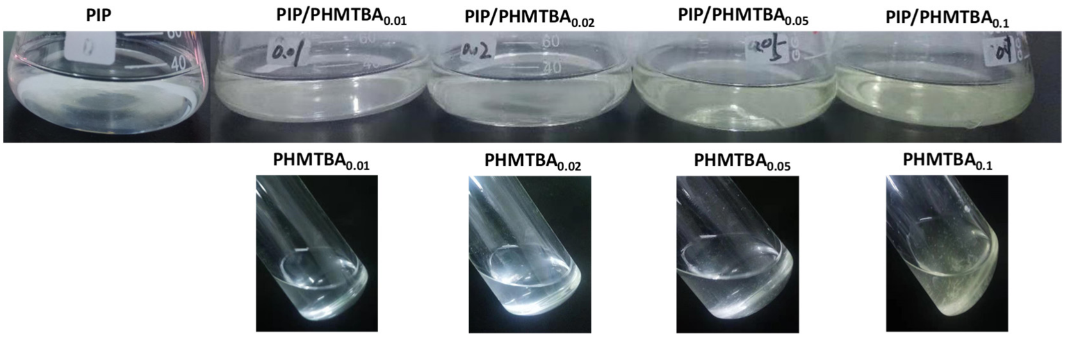

Figure S4. Photos of PIP/PHMTBA aqueous phase solution.

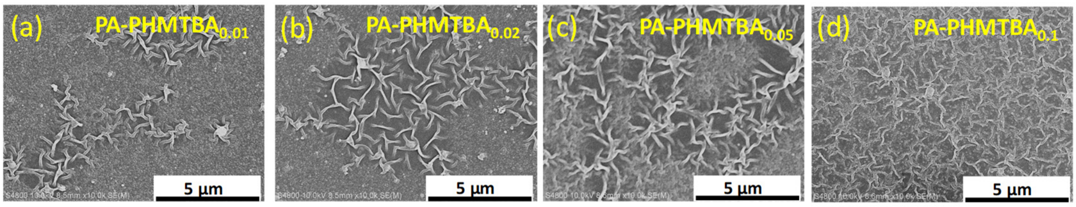

Figure S5. (a-d) Local SEM surface images of PHMTBA-modified TFC membranes.

Table S1. The hydrated radius of different ions [1].

Ion	Hydrated radius (nm)
Na^+	0.36
Mg^{2+}	0.43
Cl^-	0.33
SO_4^{2-}	0.38

Reference

[1] A.G. Volkov.; S. Paula.; D.W. Deamer. Two mechanisms of permeation of small neutral molecules and hydrated ions across phospholipid bilayers. *Bioelectroch. Bioener* 1997, 42, 153-160.
